# Outcomes of non-vitamin K oral anticoagulants for secondary prevention in ischemic stroke with atrial fibrillation

**DOI:** 10.1038/s41598-024-60660-z

**Published:** 2024-04-29

**Authors:** Ki-Woong Nam, Hyung-Min Kwon, Yong-Seok Lee, Sung-Ho Won, Hye-Sung Moon, Jong-Ho Park

**Affiliations:** 1 Department of Neurology, Seoul Metropolitan Government-Seoul National University Boramae Medical Center, 20 Boramae-ro 5-gil, Dongjak-Gu, Seoul, 07061 South Korea; 2 Department of Neurology, Seoul National University College of Medicine, Seoul, South Korea; 3 Department of Public Health Sciences, Graduate School of Public Health, Seoul National University, Seoul, South Korea; 4 Department of Neurology, Samsung Changwon Hospital, Sungkyunkwan University School of Medicine, Changwon, South Korea

**Keywords:** Neurology, Neurological disorders

## Abstract

Previous studies have rarely investigated the role of non-vitamin K oral anticoagulants (NOAC) and warfarin in the secondary prevention of ischemic stroke patients with nonvalvular atrial fibrillation (NVAF). In this study, we compared the effectiveness and safety of NOAC and warfarin for secondary prevention in Korean ischemic stroke patients with NVAF. Based on the Korean National Health Insurance Service Database, this study included 21,064 oral anticoagulants-naïve acute ischemic stroke patients with NVAF between July 2015 and June 2019. The main study outcomes included ischemic stroke, systemic embolism, major bleeding, and death. During the observational periods, NOAC users had a significantly decreased risk of ischemic stroke + systemic embolism (adjusted hazard ratio [aHR] 0.86; 95% confidence interval [CI] 0.78–0.95), ischemic stroke (aHR 0.89; 95% CI 0.81–0.99), major bleeding (aHR 0.78; 95% CI 0.68–0.89), and all-cause death (aHR 0.87; 95% CI 0.81–0.93). Standard-dose NOAC users had a lower risk of ischemic stroke, systemic embolism, and major bleeding events than warfarin users. In contrast, low-dose NOAC users did not differ in risk from warfarin users for all outcomes. In conclusion, NOACs were associated with a lower risk of secondary thromboembolic events and bleeding complications in Korean ischemic stroke patients with NVAF than warfarin.

## Introduction

Atrial fibrillation (AF) has increased rapidly with the increase in aging population worldwide^1,2^. Consequently, thromboembolic complications related to AF have emerged as major health issues^3,4^. AF is a major risk factor for stroke, and AF-related stroke has a more severe and fatal prognosis than strokes caused by other mechanisms^3,5,6^. For primary and secondary prevention of stroke in patients with AF, warfarin has been used as a standard treatment to effectively reduce thromboembolic risk. However, long-term use of warfarin increases the risk of bleeding, and it is difficult to maintain a therapeutic range^1,7^.

To date, several pivotal randomized controlled trials (RCTs) have proven that non-vitamin K oral anticoagulants (NOACs) are more effective and safer than warfarin in the primary prevention of ischemic stroke in patients with nonvalvular AF (NVAF)^8–11^. Based on these data, several guidelines in the US and Europe recommend that NOAC should be preferentially used in NVAF patients as a gold standard^12–14^. In addition, several observational studies of various groups, including the Korean population, have demonstrated that NOACs are superior to warfarin even in real-world setting^15–17^.

Stroke is a disease that requires as much attention to recurrence as occurrence; however, there is a scarcity of RCTs or observational studies dealing with the effects of NOAC on the secondary prevention of stroke. Of course, subgroup analysis or meta-analysis of pivotal RCT participants have repeatedly shown that NOAC is more effective and safer than warfarin for secondary prevention of thromboembolic events in patients with a history of stroke or transient ischemic attack (TIA)^18–23^. However, these studies were originally designed to evaluate the primary prevention of stroke, and acute or severe stroke patients were excluded. Therefore, the results did not accurately reflect the real-world outcomes experienced by acute ischemic stroke (AIS) patients with NVAF during treatment.

In this nationwide population-based study, we compared the effectiveness and safety of NOAC and warfarin for secondary prevention of thromboembolic complications in AIS patients with NVAF who used oral anticoagulants for the first-time during hospitalization.

## Results

### Study population

**Figure 1:**
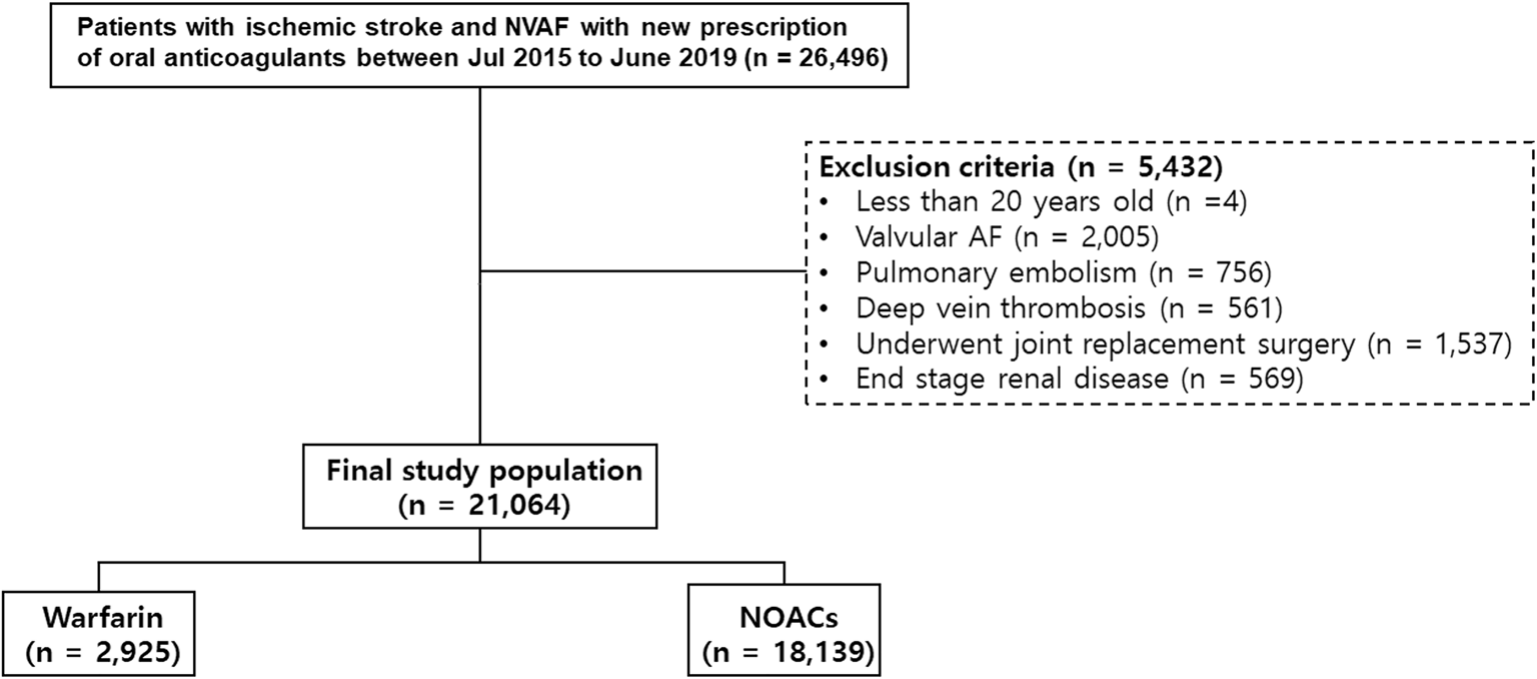
Flowchart of the study population. *NVAF* nonvalvular atrial fibrillation, *AF* atrial fibrillation, *NOACs* non-vitamin K oral anticoagulants.

Initially, the cohort included 26,496 AIS patients with NVAF who initiated OAC therapy between July 2015 and June 2019. Among them, 21,064 novel users of NOACs or warfarin met the inclusion and exclusion criteria, including 2925 (13.9%) patients who received warfarin and 18,139 (86.1%) who received NOACs (Fig. 1).

The baseline characteristics of the patients according to the treatment type are shown in Table 1. Compared to patients receiving warfarin, those receiving NOACs were older (70.66 ± 13.20 vs. 75.29 ± 10.69, *P* < 0.001), had a lower proportion of males (57.9% vs. 55.2%, *P* = 0.008), higher CHA_2_DS_2_-VASc scores (5.54 ± 1.64 vs. 5.81 ± 1.56, *P* < 0.001), a higher likelihood of hypertension (73.6% vs. 75.5%, *P* = 0.032) and peripheral artery disease (22.0% vs. 24.3%, *P* = 0.008), and a lower likelihood of prior myocardial infarction (5.5% vs. 4.1%, *P* < 0.001) and renal disease (16.1% vs. 11.6%, *P* < 0.001). Considering the number of patients between the two groups, we performed a 1:4 propensity score matching according to the treatment groups (Supplementary Table 2). After adjusting for propensity score matching, the clinical covariates were well-balanced (Supplementary Fig. 1).

**Table 1:** Baseline characteristics before and after 1:4 propensity score matching by treatment group (warfarin versus NOACs).

	Before propensity score matching				After propensity score matching			
	Warfarin (n = 2925)	NOACs (n = 18,139)	*P* value	ASD	Warfarin (n = 2889)	NOACs (n = 14,843)	*P* value	ASD
Age, y			< 0.001	0.432			< 0.001	0.007
Mean ± SD	70.66 ± 13.20	75.29 ± 10.69			71.08 ± 12.69	73.68 ± 10.67		
Median (IQR)	73 [62–80]	77 [69–83]			74 [62–80]	75 [67–81]		
Male sex	1693 (57.9)	10,020 (55.2)	0.008	0.053	1672 (57.9)	8422 (56.7)	0.269	0.002
BMI, kg/m^2^			0.353	0.018			0.629	0.010
Mean ± SD	24.11 ± 3.07	24.16 ± 3.12			24.10 ± 3.07	24.13 ± 3.10		
Median (IQR)	24.08 [22.30–25.80]	24.00 [22.40–25.80]			24.08 [22.30–25.80]	24.03 [22.40–25.70]		
CHA_2_DS_2_-VASc score			< 0.001	0.170			< 0.001	0.007
Mean ± SD	5.54 ± 1.64	5.81 ± 1.56			5.56 ± 1.64	5.72 ± 1.60		
Median (IQR)	6 [4–7]	6 [5–7]			6 [4–7]	6 [5–7]		
Hypertension	2153 (73.6)	13,689 (75.5)	0.032	0.043	2137 (74.0)	11,163 (75.2)	0.167	0.001
Diabetes mellitus	883 (30.2)	5265 (29.0)	0.207	0.025	878 (30.4)	4443 (29.9)	0.639	0.003
Dyslipidemia	2468 (84.4)	15,319 (84.5)	0.937	0.002	2445 (84.6)	12,587 (84.8)	0.839	0.003
Heart failure	897 (30.7)	5410 (29.8)	0.368	0.018	884 (30.6)	4481 (30.2)	0.677	0.005
Prior MI	162 (5.5)	743 (4.1)	< 0.001	0.067	155 (5.4)	683 (4.6)	0.085	0.001
PAD	643 (22.0)	4398 (24.3)	0.008	0.067	643 (22.3)	3466 (23.4)	0.211	0.002
COPD	597 (20.4)	3578 (19.7)	0.403	0.017	589 (20.4)	3009 (20.3)	0.908	0.001
Renal disease	471 (16.1)	2110 (11.6)	< 0.001	0.130	456 (15.8)	1980 (13.3)	0.001	0.001

*NOAC* non-vitamin K oral anticoagulants, *BMI* body mass index, *MI* myocardial infarction, *PAD* peripheral artery disease, *COPD* chronic obstructive pulmonary disease.

Values are n (%), unless otherwise indicated.

### Clinical outcomes

**Figure 2:**
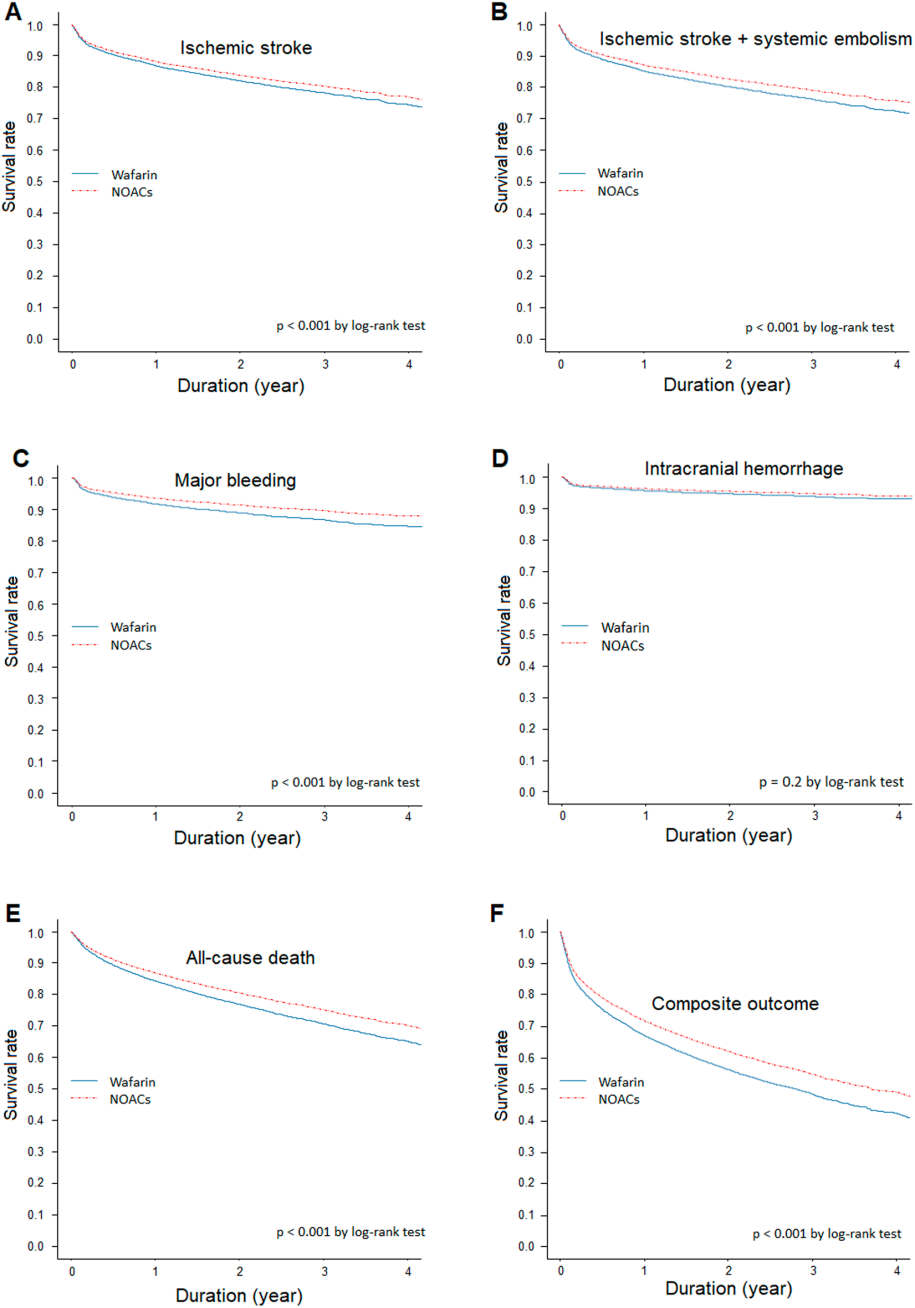
Cumulative incidence of effectiveness and safety outcomes in NOACs and matched warfarin groups. Compared to warfarin, NOAC users had a significantly lower risk of ischemic stroke (**A**), ischemic stroke + systemic embolism (**B**), major bleeding (**C**), all-cause death (**E**), and composite outcome of ischemic stroke + systemic embolism + major bleeding + all-cause death (**F**). However, in the case of ICH, no statistically significant difference was observed between the two groups (**D**).

**Figure 3:**
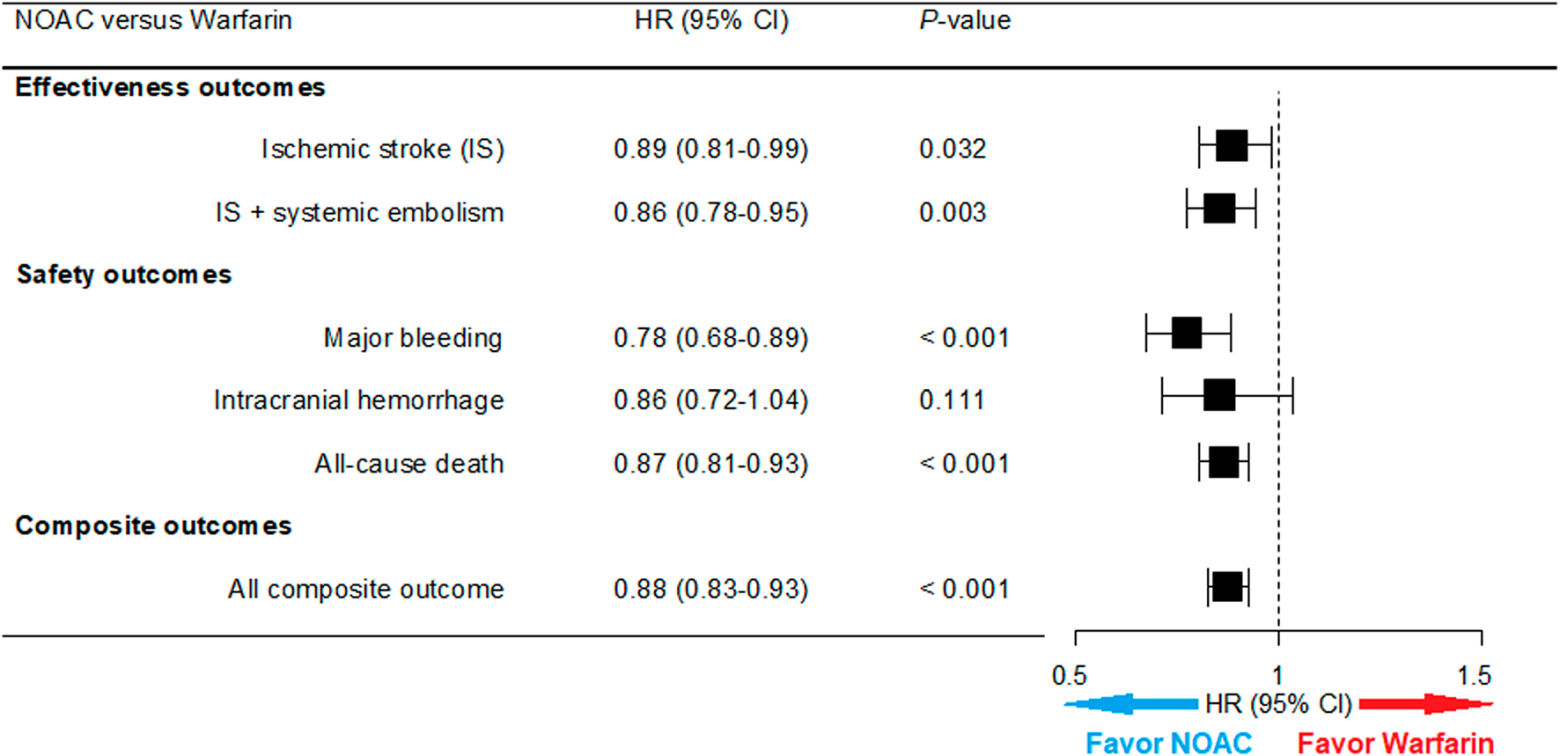
NOACs versus warfarin groups: Hazard ratios of six outcomes. Compared with warfarin user as the reference, NOACs were associated with an 11%, 14%, 22%, and 13% risk reduction in ischemic stroke, ischemic stroke + systemic embolism, major bleeding, and all-cause death, respectively. In addition, NOAC user had a significantly lower risk of composite outcome of ischemic stroke + systemic embolism + major bleeding + all-cause death (hazard ratio 0.88; 95% confidence interval 0.83–0.93).

The median follow-up period for the cohort was 15.5 months. The number of events and incidence rates of the effectiveness and safety outcomes are summarized in Supplementary Table 3. Compared to the warfarin group, patients receiving NOACs showed a lower annual incidence rate of primary effectiveness (9.65 vs. 9.67, per 100 person-years) and safety outcomes (4.58 vs. 4.99, per 100 person-years). Figure 2 shows the weighted cumulative incidence curves for the six outcomes by the treatment group. The aHR for the effectiveness and safety outcomes of NOACs and warfarin are presented in Fig. 3 and Supplementary Tables 4–9. NOAC user was significantly associated with a lower risk of ischemic stroke (aHR 0.89; 95% CI 0.81–0.99), ischemic stroke + systemic embolism (aHR 0.86; 95% CI 0.78–0.95), major bleeding (aHR 0.78; 95% CI 0.68–0.89), all-cause death (aHR 0.87; 95% CI 0.81–0.93), and composite outcomes (aHR 0.88; 95% CI 0.83–0.93). Regarding risk associated with ICH, NOAC users showed no statistically significant difference from warfarin users (aHR 0.86; 95% CI 0.72–1.04).

### Comparisons according to NOAC dose

Among 14,843 NOAC users who completed propensity score matching, 6860 (46.2%) patients used a standard-dose and 7983 (53.8%) patients used a low-dose. Table 2 shows a comparison of baseline characteristics between patients using warfarin and those using standard- and low-dose NOACs. Patients using low-dose NOAC were the oldest, had the lowest body mass index, the highest CHA_2_DS_2_-VASc scores, and the highest likelihood of comorbidities. In contrast, standard-dose NOAC users were the youngest, had the lowest CHA_2_DS_2_-VASc scores, and the lowest prevalence of most comorbidities, compared to warfarin or low-dose NOAC users.

**Table 2:** Baseline characteristics of warfarin, low-dose and standard-dose NOAC groups.

	Warfarin (n = 2889)	Low-dose NOAC (n = 7983)	Standard-dose NOAC (n = 6860)	*P* value
Age, y				< 0.001
Mean ± SD	71.08 ± 12.69	76.98 ± 9.87	68.84 ± 10.27	
Median (IQR)	74 [62–80]	79 [72–84]	71 [63–77]	
Male sex	1672 (57.9)	3872 (48.5)	4550 (66.3)	< 0.001
Body mass index, kg/m^2^				< 0.001
Mean ± SD	24.10 ± 3.07	23.78 ± 3.03	24.55 ± 3.13	
Median (IQR)	24.08 [22.30–25.80]	23.79 [22.10–25.30]	24.40 [22.80–26.20]	
CHA_2_DS_2_-VASc score				< 0.001
Mean ± SD	5.56 ± 1.64	6.13 ± 1.48	5.25 ± 1.60	
Median (IQR)	6 [4–7]	6 [5–7]	5 [4–6]	
Hypertension	2137 (74.0)	6275 (78.6)	4888 (71.3)	< 0.001
Diabetes mellitus	878 (30.4)	2547 (31.9)	1896 (27.6)	< 0.001
Dyslipidemia	2445 (84.6)	6770 (84.8)	5817 (84.8)	0.973
Heart failure	155 (5.4)	439 (5.5)	244 (3.6)	< 0.001
Prior myocardial infarction	643 (22.3)	1992 (25.0)	1474 (21.5)	< 0.001
Peripheral artery disease	884 (30.6)	2647 (33.2)	1834 (26.7)	< 0.001
Chronic obstructive pulmonary disease	589 (20.4)	1759 (22.0)	1250 (18.2)	< 0.001
Renal disease	456 (15.8)	1336 (16.7)	644 (9.4)	< 0.001

*NOAC* non-vitamin K oral anticoagulant.

Values are n (%), unless otherwise indicated.

**Figure 4:**
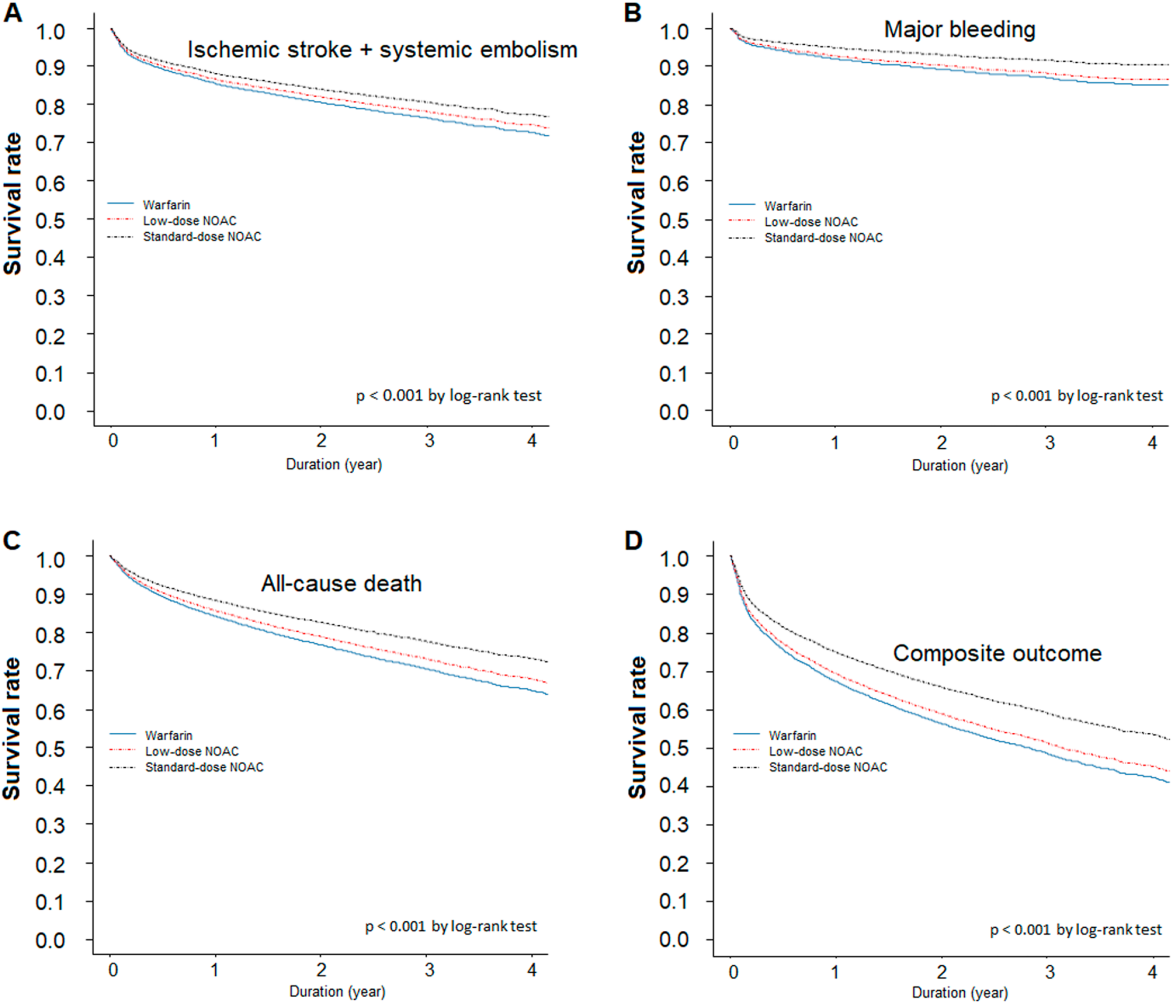
Cumulative incidence of effectiveness and safety outcomes in low- and standard-NOACs and matched warfarin groups. Compared to warfarin, standard-dose NOAC users had a significantly lower risk for ischemic stroke + systemic embolism (**A**), major bleeding (**B**), all-cause death (**C**), and composite outcome of ischemic stroke + systemic embolism + major bleeding + all-cause death (**D**). Low-dose NOAC users did not differ in risk from warfarin users in all of the four outcomes.

Compared with the warfarin group, standard-dose NOAC users showed a lower annual event rate of primary effectiveness and safety outcomes, all-cause death, and composite outcomes. In contrast, low-dose NOAC users showed higher annual incidence rates than warfarin users in all four outcomes (Supplementary Table 10). Figure 4 shows the weighted cumulative incidence curves for the primary effectiveness and safety outcomes, all-cause death, and composite outcomes between warfarin and standard- and low-dose NOAC users. Standard-dose NOAC user was significantly associated with a lower risk of primary effectiveness (aHR 0.81; 95% CI 0.73–0.90) and safety (aHR 0.66; 95% CI 0.56–0.77) outcomes, all-cause death (aHR 0.73; 95% CI 0.67–0.79), and composite outcomes (aHR 0.76; 95% CI 0.71–0.82). In contrast, low-dose NOAC users showed no significant difference in risk compared to warfarin users in all of the four outcomes (Supplementary Table 11).

## Discussion

This nationwide population-based cohort study showed that NOACs were superior to warfarin for the secondary prevention of thromboembolic events in Korean ischemic stroke patients with NVAF. In addition, although the risk of ICH was not statistically different, NOACs were overall safer than warfarin. Compared to warfarin, standard-dose NOAC showed superior secondary prevention effectiveness and safety, whereas low-dose NOAC had no beneficial effect over warfarin.

We used data from the Korean NHIS to analyze the real-world data. With its high guarantee and reasonable price, the Korean NHIS currently covers the majority of the population. During any medical activity, physicians enter the disease and treatment codes to receive support from the state for the reimbursement of the medical item, and the data is entered into the NHIS database. After all, the Korean NHIS contains the complete medical history of an individual until the occurrence of clinical events (e.g., stroke) or death. Therefore, data loss was minimized, and complete follow-up of the study population was possible during the observation period. In addition, the use of NOAC for the prevention of thromboembolic complications in patients with NVAF has been fully reimbursed in Korea since July 2015, and the use of NOAC has increased rapidly^24^. Therefore, we believe that the results of our study population, which were included from July 2015 to June 2019, are relatively recent real-world data that well reflect the selection of OACs for secondary prevention in ischemic stroke patients with NVAF and their prognosis.

In addition, our study reflects real-world data including relatively diverse clinical situations. As mentioned earlier, previous RCTs have demonstrated the primary prevention effectiveness and safety of four types of NOACs^8–11^. Subgroup and meta-analysis studies performed based on the data of RCTs have demonstrated that NOAC was superior to warfarin even in secondary prevention^18–21^. However, these RCTs were conducted on well-selected patients and were not originally designed to evaluate the secondary prevention effectiveness of NOACs. The study population of these analyses included patients with a history of stroke or TIA from among those enrolled in the RCTs. However, with the exception of the ROCKET AF trial (52%), only 19–28% of the patients enrolled in the remaining three RCTs had a history of stroke or TIA^7,25^. Further, recent strokes that occurred within 14 days and severe/disabling strokes were excluded from the analysis. These high-risk groups and periods with poor prognoses and high recurrence rates should be prioritized to get relevant information. A recent observational study compared the real-world data on secondary prevention of NOAC and warfarin using NHIS data in Korea; however, the study population was still defined using the history of stroke^5^. As a result, even though “recent stroke” was defined as a stroke that occurred within 6 months, only 6499 of the 61,568 subjects analyzed were classified as recent stroke patients^5^. In addition, in selecting the study population, the authors defined index stroke only using past history without confirmatory brain imaging. Therefore, the results of that study were analyzed from the perspective of NVAF patients rather than stroke patients. In contrast, our study included AIS patients with NVAF as the study population and defined the time when they first used NOAC or warfarin during hospitalization as the time of enrolment. Based on this, we attempted to select an OAC for the first time after AIS occurred in NVAF patients, and to reflect the secondary prevention effectiveness and safety that occurred thereafter.

In previous pivotal RCTs, Asian participants accounted for only 10–15% of the study population^8–11^. Therefore, it was necessary to verify whether the results of these studies are applicable to Asians despite racial differences, which was confirmed by our study results. However, our findings related to the NOAC dosage should be carefully interpreted. Asians have lower body mass index and renal clearance than whites^26–28^. For these reasons, Asians show different pharmacokinetics from whites, and some studies report that Asian low-dose NOAC users and white standard-dose NOAC users maintain similar drug concentrations and have similar clinical effects^29,30^. In addition, it is said that Asians have a relatively high risk of bleeding complications and do not maintain the therapeutic range of warfarin well^26,31^. In fact, according to recent Korean data, even for patients intensively managed with short-term follow-up at large university hospitals, the period during which the warfarin concentration was within the therapeutic range was only 49.1% of the total period^32^. Consequently, for Asians, the use of NOAC, especially low-dose NOAC, seems to have merit. In reality, many Asian doctors have stated that because of the risk of bleeding complications, more people are being prescribed low-dose NOACs than they are eligible for, resulting in 18.8% of the Asian population being undertreated^33,34^.

Even in our study, more than half (53.8%) of the 14,843 NOAC users used low-dose NOACs. However, low-dose NOAC users were inferior to standard-dose NOAC users in terms of both secondary prevention effectiveness and safety, and there was no significant difference when compared with warfarin users. This was because low-dose NOAC users are significantly older, have lower body mass index and higher CHA_2_DS_2_-VASc scores, and have various comorbidities^25,35^. As a result, even though the NOAC dose was low, bleeding complications, including ICH, would have been more likely. In addition, the use of low-dose NOAC without guideline adherence might not sufficiently prevent thromboembolic complications, resulting in a decrease in secondary prevention effectiveness^36^. As a result, as shown in Supplementary Table 11, low-dose NOACs showed no advantage over warfarin. On the other hand, standard-dose users showed superiority in safety as well as effectiveness compared to warfarin. Consequently, our findings once again demonstrate the importance of selecting adherent NOAC doses based on the guidelines.

Our study has several limitations. First, this was a non-randomized, retrospective, observational study. Therefore, there may be some degree of selection or ascertainment bias. Moreover, even if propensity score matching was performed during the analysis process, there may be an effect of an unknown confounding factor. Second, this study was conducted based on Korean NHIS data, a national administrative data record. Therefore, although probably not significant, the effects of coding errors or missing data should be considered. Third, detailed radiological findings related to ischemic stroke were not included in the analysis. In addition to the variables included, radiological findings, such as stroke lesion pattern, intracranial/extracranial atherosclerosis, and cerebral microbleeds, may influence the incidence of stroke recurrence or hemorrhagic complications in NOAC users^37,38^. However, since we used an already constructed national database, it was difficult to retrospectively add the desired data. Fourth, we provided information on the embolic risk of the entire study population through the CHA_2_DS_2_-VASc score. However, it would have been more helpful in interpreting the results if information on bleeding risk was also provided through the HAS-BLED score. Finally, we do not know whether warfarin users maintained their drug concentrations adequately within the therapeutic range during the observation period. As described above, warfarin is affected by various factors compared with NOAC, and it is difficult to maintain an appropriate drug concentration in the body. However, this study was not an RCT that compared superiority and inferiority between drugs under the same conditions, but rather a study that analyzed real-world data experienced by patients in real situations. Therefore, we believe that the effect of this unstable warfarin concentration on the body was also included in the analysis.

In conclusion, NOAC use was associated with a lower risk of secondary thromboembolic events and bleeding complications in ischemic stroke patients with NVAF. Patients using low-dose NOAC did not differ from warfarin users in both secondary prevention effectiveness and safety^5,28^. On the other hand, standard-dose NOAC users were superior to warfarin users in all outcomes. Therefore, the use of an appropriate NOAC dose that adheres to the guidelines for each individual seems to be important for the secondary prevention of ischemic stroke.

## Methods

The clinical data for this study were obtained from the nationwide administrative health claims database established by the Korean National Health Insurance Service (NHIS). The NHIS is a mandatory health insurance service that provides comprehensive medical care to ~ 97% of the Korean population^39^. The remaining 3% comprise the lowest-income bracket, who are covered by the government-financed Medical Aid program managed by NHIS. The NHIS database contains comprehensive claims data for medical services and costs, including diagnosis, treatment, procedure, hospital admission and discharge, and prescription records. All diagnostic data were coded according to the International Classification of Disease, 10th Revision, Clinical Modification (ICD-10-CM) codes. Information about dispensed prescriptions (e.g., type of medication, pill number, dosage, and days supplied) was obtained from prescription claims data. On the basis of this dataset, we evaluated the demographic and clinical factors necessary for this study.

The Institutional Review Board (IRB) of the Seoul Metropolitan Government-Seoul National University Boramae Medical Center approved this study (IRB Number: 07-2021-14). The requirement for informed consent was waived by the IRB of the Seoul Metropolitan Government-Seoul National University Boramae Medical Center because the data were publicly available and anonymized under the confidentiality guidelines. All experiments were performed in accordance with the Declaration of Helsinki and the relevant guidelines.

### Study population

The study cohort comprised AIS patients with NVAF who were taking oral anticoagulants (OACs) for the first time between July 2015 and June 2019. To select an accurate study population suitable for the study hypothesis, we defined AIS patients with NVAF as those who met all of the following conditions: (1) ICD-10-CM codes for both ischemic stroke and NVAF; (2) hospitalization during the study period; and (3) underwent brain magnetic resonance imaging (MRI) just before or during hospitalization. Among them, the following patients were excluded according to the exclusion criteria: (1) age < 20 years; (2) prior use of warfarin or NOAC before the start of the study period; (3) valvular AF (e.g., severe mitral stenosis or prosthetic valve); (4) prior history of pulmonary thromboembolism or deep vein thrombosis; (5) prior joint replacement surgery; and (6) undergoing renal replacement therapy^40^. Finally, 21,064 OAC-naive AIS patients with NVAF were included in the analysis.

### Covariates

The baseline demographic characteristics and comorbidities were extracted from the database. Comorbidities were defined according to ICD-10-CM codes within 1 year of cohort entry, and detailed definitions are summarized in Supplementary Table 1. Based on previous studies and literatures, and clinical significance, hypertension, diabetes mellitus, dyslipidemia, heart failure, prior myocardial infarction, peripheral artery disease, chronic obstructive pulmonary disease, and renal disease were evaluated as comorbidities. The CHA_2_DS_2_-VASc score was calculated by summing the scores of each item according to the formula (age ≥ 75 years, 2 points; age 65–74 years, 1 point; female sex, 1 point; hypertension, 1 point; diabetes mellitus, 1 point; heart failure, 1 point; history of stroke or TIA, 2 points; vascular disease, 1 point).

The main independent variable in this study was OAC. All patients included in the study used warfarin or NOAC within the acute period during hospitalization after the index stroke. NOACs included four classes: dabigatran, rivaroxaban, apixaban, and edoxaban. Based on standard recommendations for ischemic stroke prevention, standard-dose NOACs (e.g., dabigatran 150 mg bid, rivaroxaban 20 mg qd, apixaban 5 mg bid, edoxaban 60 mg qd) and low-dose NOACs (e.g., dabigatran 110 mg bid, rivaroxaban 15 mg qd, apixaban 2.5 mg bid, edoxaban 30 mg qd) were defined. In the case of dabigatran, both 110 mg bid and 150 mg bid are standard-dose by definition, but in this study, 110 mg bid was classified as low-dose NOAC for analysis.

### Outcomes and follow-up

The primary effectiveness outcome of this study was the composite of ischemic stroke and systemic embolism, and the primary safety outcome was major bleeding. Major bleeding was defined as a composite variable of intracranial hemorrhage (ICH) and gastrointestinal (GI) bleeding. In addition, ischemic stroke was used as a secondary effectiveness outcome and ICH and all-cause death as secondary safety outcomes. For accurate analysis, brain imaging was essential in the definition of ischemic stroke recurrence and ICH, such as index stroke. The detailed definitions of each outcome are presented in Supplementary Table 1. In addition, to compare superiority and inferiority between drugs, a composite outcome including all effectiveness and safety outcomes was defined as one outcome variable.

The time point of patient enrolment was based on the day they first received a prescription for OACs after admission. The observation period lasted until December 2019, and patients were censored at the end of the observation period or the occurrence of outcome events or death during the observation period. The occurrence and date of the patient outcome events were confirmed using the National Population Registry of the Korea National Statistical Office.

### Statistical analysis

Baseline characteristics and clinical outcomes of warfarin and NOAC users were evaluated. Continuous variables with normal distribution are expressed as mean ± SD, and those without normal distribution are presented as median + interquartile range. Categorical variables are presented as frequencies with percentages. Differences between treatment groups were analyzed using Student’s t-test or Mann–Whitney U-test for continuous variables and chi-squared tests or Fisher’s exact test for categorical variables.

To minimize the effects of confounding factors and residual selection bias between the two treatment groups, a propensity score matching method was applied. Warfarin users and NOAC users were matched 1:4 based on the propensity scores calculated by logistic regression model which included age, sex, body mass index, CHA_2_DS_2_-VASc score, hypertension, diabetes mellitus, dyslipidemia, heart failure, myocardial infarction, peripheral artery disease, chronic obstructive pulmonary disease, and renal disease (Supplementary Table 2). The nearest neighbor matching method without replacement with a caliper of 0.05 was used to match the patients. The balance of covariates was checked based on the absolute standardized mean difference after matching (Supplementary Fig. 1).

The frequency of effectiveness and safety outcomes is presented as the incidence rate per 100 person-years. The incidence rate was calculated based on the number of event outcomes, number of observed subjects, and observation period of each subject. The cumulative events of each clinical outcome were assessed using Kaplan–Meier analysis, and comparisons between treatment groups were performed using the log-rank test. Considering the influence of various confounding factors, multivariable cox regression analysis was used to compare the differences in risk reduction on the effectiveness and safety outcomes between the two treatment groups. The warfarin user was set as a reference and the adjusted hazard ratio (aHR) and 95% confidence interval (CI) of the NOAC users were calculated, which indicated the relative risk reduction difference between the two treatment groups.

To compare the relative secondary prevention effectiveness and safety with warfarin according to the dosage of NOAC, the NOAC users were divided into standard-dose NOAC users (dabigatran 150 mg bid, rivaroxaban 20 mg qd, apixaban 5 mg bid, edoxaban 60 mg qd) and low-dose NOAC users. The baseline characteristics of the three treatment groups were compared, and clinical outcomes were compared using Kaplan–Meier analysis and cox regression analysis. Similarly, the relative risk reduction of standard-dose and low-dose NOAC users was evaluated, with warfarin users as a reference.

All statistical analyses were performed using SAS, version 9.4 (SAS Inc., Cary, NC, USA) and R statistical software version 4.0.3 (R Foundation for Statistical Computing, Vienna, Austria). In this study, all tests were two-sided and variables with *P* < 0.05 were considered statistically significant.

### Supplementary Information

Supplementary Information.

## Data Availability

Data from the Korean NHIS can be accessed via the Health Insurance Data Service website (http://nhiss.nhis.or.kr). For data access, researchers must submit their study proposals for approval from each institutional review board, which is reviewed by the NHIS review committee. Raw data could not be retrieved from the NHIS server. Further enquiries can be directed to the corresponding author (H.-M.K.).
