# Supplementary material for: Outcomes of non-vitamin K oral anticoagulants for secondary prevention in ischemic stroke with atrial fibrillation
Source: Sci Rep. 2024 Apr 29;14:9838. doi: 10.1038/s41598-024-60660-z (PMC11058194; doi:10.1038/s41598-024-60660-z)
Supplement: Supplementary file 1 — Supplementary Information. [file 41598_2024_60660_MOESM1_ESM.pdf]

Supplemental Materials

Supplementary Figure 1. Distribution of propensity scores in NOACs and warfarin group and balance assessment after matching

A. Propensity score distribution before and after matching

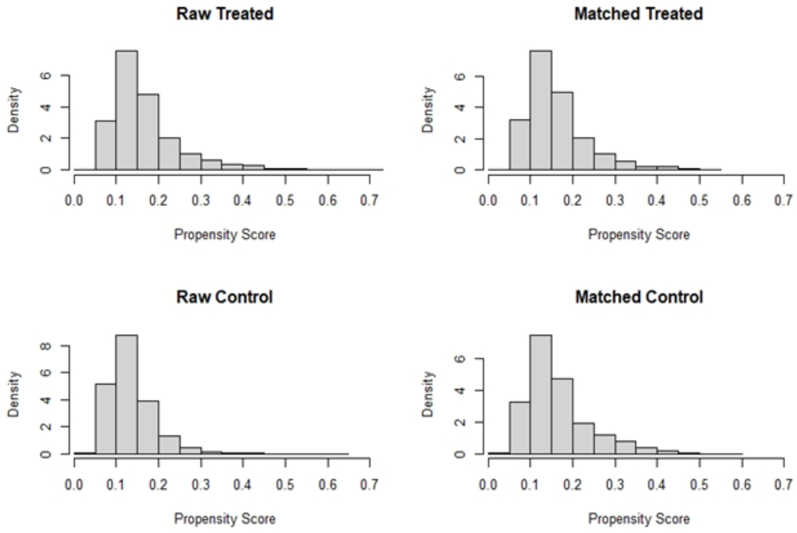

B. Balance assessment after matching

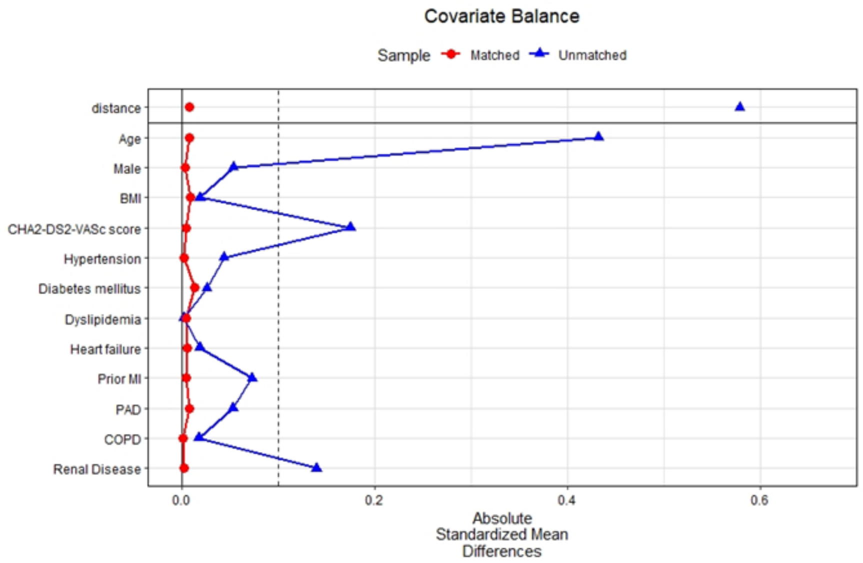

Supplementary Table 1. Definition of variables

Variables	ICD-10-CM code	Additional definition
Atrial fibrillation	I480-I484, I489	Admission or outpatient department ≥ 1
Ischemic stroke	I63	Admission ≥ 1<br>Brain imaging (MRI) ≥ 1
Valvular atrial fibrillation	I050, I052, I059, Z952-Z954	-
Pulmonary embolism	I26	Admission ≥ 1
Deep vein thrombosis	I802	Admission ≥ 1
Received joint replacement operation	NO711, N1711, N1721, N2070, N3710, N3721, N3717, N3720, N2072, N2077, N3722, N3727	Admission ≥ 1
End stage renal disease	N18.5, Z49	Dialysis ≥ 2
Systemic embolism	I74	Admission ≥ 1
Intracranial hemorrhage	I60-I62	Admission ≥ 1 or RBC transfusion ≥ 1
Gastrointestinal bleeding	K22.6, K25.0, K25.2, K25.4, K25.6, K26.0, K26.2, K26.4, K26.6, K27.0, K27.2, K27.4, K27.6, K28.0, K28.2, K28.4, K28.6, K29.0, K62.5, K92.0, K92.1, K92.2	Admission ≥ 1 or RBC transfusion ≥ 1
Major bleeding	-	Intracranial bleeding or gastrointestinal bleeding
Hypertension	I10-I13, I15	Admission ≥ 1 or outpatient department ≥ 2<br>Minimum 1 prescription of anti-hypertensive agents
Diabetes mellitus	E11-E14	Admission ≥ 1 or outpatient department ≥ 2<br>Minimum 1 prescription of anti-diabetic agents
Dyslipidemia	E78	Admission ≥ 1 or outpatient department ≥ 1
Heart failure	I50	Admission ≥ 1 or outpatient department ≥ 1
Myocardial infarction	I21, I22	Admission ≥ 1 or outpatient department ≥ 1
Peripheral artery disease	I70, I73	Admission ≥ 1 or outpatient department ≥ 2

Chronic obstructive pulmonary disease	J41-J44	Admission ≥ 1 or outpatient department ≥ 1
Renal disease	I13.1, N03, N05, N10-N19, Z49, Z94.0, Z99.2	Admission ≥ 1 or outpatient department ≥ 1

Supplementary Table 2. The logistic regression model used to calculate the propensity score matching

	Coefficient	SE	OR (95% CI)	P value
**Age (per year)**	-0.050	0.003	0.951 (0.945 to 0.956)	< 0.001
**Male Sex**	0.000	0.056	1.000 (0.897 to 1.115)	0.999
**BMI (per kg/m^2)**	-0.036	0.007	0.965 (0.952 to 0.978)	< 0.001
**CHA_2DS_2-VASc score (per point)**	0.158	0.034	1.171 (1.097 to 1.251)	< 0.001
**Hypertension**	-0.045	0.053	0.956 (0.861 to 1.062)	0.405
**Diabetes mellitus**	-0.063	0.050	0.939 (0.851 to 1.036)	0.208
**Dyslipidemia**	-0.063	0.057	0.939 (0.840 to 1.050)	0.272
**Heart failure**	-0.136	0.058	0.873 (0.778 to 0.979)	0.020
**Prior myocardial infarction**	0.156	0.095	1.169 (0.969 to 1.409)	0.102
**Peripheral artery disease**	-0.218	0.060	0.804 (0.715 to 0.904)	< 0.001
**Chronic obstructive pulmonary disease**	0.098	0.051	1.103 (0.998 to 1.220)	0.055
**Renal disease**	0.461	0.058	1.585 (1.415 to 1.775)	< 0.001

Supplementary Table 3. Number of composite and safety outcomes by treatment group (warfarin versus NOACs)

	Warfarin		NOACs	
	Event (%)	IR	Event (%)	IR
Ischemic stroke	465 (16.1)	8.63	1,908 (12.9)	8.87
Ischemic stroke + systemic embolism	514 (17.8)	9.67	2,059 (13.9)	9.65
Major bleeding	281 (9.7)	4.99	1,026 (6.9)	4.58
Intracranial hemorrhage	144 (5.0)	2.49	577 (3.9)	2.53
All-cause death	915 (31.7)	15.28	3,754 (25.3)	15.98
Ischemic stroke + systemic embolism + GI bleeding + intracranial hemorrhage + all-cause death	1,362 (47.1)	27.01	5,728 (38.6)	27.99

IR: indicated incidence rate per 100 Person-year

Supplementary Table 4. Univariate and multivariable cox regression analysis for ischemic stroke in patients using warfarin versus NOACs

	Univariate analysis		Multivariable analysis	
	Crude HR (95% CI)	*P*-value	Adjusted HR (95% CI)	*P*-value
Type of anticoagulants				
Warfarin	1 (Ref)		1 (Ref)	
NOACs	0.91 (0.82-1.00)	0.058	0.89 (0.81-0.99)	0.032
Age, years				
< 65	1 (Ref)		1 (Ref)	
65-74	1.15 (1.03-1.30)	0.017	1.13 (1.00-1.27)	0.051
≥ 75	1.29 (1.16-1.43)	< 0.001	1.22 (1.08-1.36)	0.001
Male sex	0.85 (0.79-0.92)	< 0.001	0.89 (0.82-0.97)	0.010
BMI, kg/m^2	1.00 (0.99-1.01)	0.929	1.00 (0.99-1.02)	0.693
Hypertension	1.07 (0.97-1.17)	0.163	0.97 (0.88-1.07)	0.579
Diabetes mellitus	1.14 (1.04-1.24)	0.004	1.10 (1.01-1.21)	0.029
Dyslipidemia	1.10 (0.98-1.24)	0.114	1.06 (0.94-1.19)	0.340
Heart failure	1.14 (1.05-1.25)	0.003	1.08 (0.99-1.18)	0.081
Prior MI	1.20 (1.00-1.44)	0.045	1.15 (0.95-1.38)	0.145
Peripheral artery disease	1.11 (1.01-1.22)	0.035	1.06 (0.96-1.16)	0.247
COPD	1.12 (1.01-1.23)	0.032	1.06 (0.96-1.18)	0.225
Renal disease	1.21 (1.08-1.36)	0.001	1.12 (0.99-1.26)	0.066

NOAC: non-vitamin K oral anticoagulants, BMI: body mass index, MI: myocardial infarction, COPD: chronic obstructive pulmonary disease

Supplementary Table 5. Univariate and multivariable cox regression analysis for ischemic stroke + systemic embolism in patients using warfarin versus NOACs

	Univariate analysis		Multivariable analysis	
	Crude HR (95% CI)	*P*-value	Adjusted HR (95% CI)	*P*-value
Type of anticoagulants				
Warfarin	1 (Ref)		1 (Ref)	
NOACs	0.88 (0.79-0.96)	0.007	0.86 (0.78-0.95)	0.003
Age, years				
< 65	1 (Ref)		1 (Ref)	
65-74	1.18 (1.06-1.33)	0.003	1.16 (1.03-1.30)	0.014
≥ 75	1.33 (1.20-1.48)	< 0.001	1.26 (1.13-1.40)	< 0.001
Male sex	0.86 (0.80-0.93)	< 0.001	0.92 (0.85-1.00)	0.038
BMI, kg/m^2	1.00 (0.99-1.01)	0.938	1.00 (0.99-1.02)	0.647
Hypertension	1.11 (1.01-1.21)	0.030	1.00 (0.91-1.10)	0.987
Diabetes mellitus	1.15 (1.06-1.25)	0.001	1.11 (1.02-1.21)	0.019
Dyslipidemia	1.10 (0.98-1.23)	0.110	1.05 (0.94-1.18)	0.406
Heart failure	1.16 (1.06-1.26)	0.001	1.09 (1.00-1.19)	0.047
Prior MI	1.18 (0.99-1.41)	0.064	1.11 (0.93-1.33)	0.248
Peripheral artery disease	1.13 (1.04-1.24)	0.006	1.08 (0.98-1.18)	0.114
COPD	1.14 (1.03-1.25)	0.009	1.07 (0.97-1.18)	0.152
Renal disease	1.26 (1.13-1.41)	< 0.001	1.16 (1.03-1.30)	0.011

NOAC: non-vitamin K oral anticoagulants, BMI: body mass index, MI: myocardial infarction, COPD: chronic obstructive pulmonary disease

Supplementary Table 6. Univariate and multivariable cox regression analysis for major bleeding in patients using warfarin versus NOACs

	Univariate analysis		Multivariable analysis	
	Crude HR (95% CI)	*P*-value	Adjusted HR (95% CI)	*P*-value
Type of anticoagulants				
Warfarin	1 (Ref)		1 (Ref)	
NOACs	0.79 (0.70-0.91)	0.001	0.78 (0.68-0.89)	< 0.001
Age, years				
< 65	1 (Ref)		1 (Ref)	
65-74	1.38 (1.16-1.63)	< 0.001	1.27 (1.07-1.51)	0.006
≥ 75	1.66 (1.43-1.93)	< 0.001	1.40 (1.19-1.65)	< 0.001
Male sex	0.83 (0.74-0.92)	0.001	0.92 (0.82-1.03)	0.144
BMI, kg/m^2	0.98 (0.96-1.00)	0.020	0.98 (0.96-1.00)	0.027
Hypertension	1.60 (1.39-1.85)	< 0.001	1.43 (1.24-1.66)	< 0.001
Diabetes mellitus	1.38 (1.23-1.54)	< 0.001	1.28 (1.14-1.44)	< 0.001
0.042Dyslipidemia	0.94 (0.81-1.10)	0.459	0.85 (0.73-0.99)	0.042
Heart failure	1.32 (1.17-1.48)	< 0.001	1.17 (1.04-1.32)	0.009
Prior MI	1.33 (1.05-1.68)	0.017	1.17 (0.92-1.48)	0.206
Peripheral artery disease	1.02 (0.90-1.16)	0.759	0.91 (0.80-1.04)	0.170
COPD	1.36 (1.20-1.54)	< 0.001	1.23 (1.08-1.40)	0.002
Renal disease	1.58 (1.37-1.83)	< 0.001	1.34 (1.16-1.55)	< 0.001

NOAC: non-vitamin K oral anticoagulants, BMI: body mass index, MI: myocardial infarction, COPD: chronic obstructive pulmonary disease

Supplementary Table 7. Univariate and multivariable cox regression analysis for intracranial hemorrhage in patients using warfarin versus NOACs

	Univariate analysis		Multivariable analysis	
	Crude HR (95% CI)	*P*-value	Adjusted HR (95% CI)	*P*-value
Type of anticoagulants				
Warfarin	1 (Ref)		1 (Ref)	
NOACs	0.85 (0.71-1.02)	0.083	0.86 (0.72-1.04)	0.111
Age, years				
< 65	1 (Ref)		1 (Ref)	
65-74	1.06 (0.87-1.30)	0.557	0.98 (0.80-1.21)	0.852
≥ 75	0.99 (0.82-1.19)	0.921	0.85 (0.70-1.04)	0.119
Male sex	0.92 (0.80-1.07)	0.291	0.90 (0.77-1.05)	0.180
BMI, kg/m^2	0.99 (0.97-1.02)	0.673	0.99 (0.96-1.01)	0.263
Hypertension	1.44 (1.20-1.74)	< 0.001	1.44 (1.19-1.74)	< 0.001
Diabetes mellitus	1.33 (1.14-1.55)	< 0.001	1.30 (1.11-1.52)	0.001
Dyslipidemia	0.87 (0.72-1.06)	0.165	0.80 (0.66-0.98)	0.029
Heart failure	1.10 (0.94-1.29)	0.232	1.03 (0.88-1.22)	0.687
Prior MI	1.21 (0.87-1.67)	0.261	1.13 (0.81-1.58)	0.459
Peripheral artery disease	0.93 (0.78-1.11)	0.445	0.88 (0.74-1.06)	0.171
COPD	1.35 (1.14-1.60)	0.001	1.33 (1.12-1.59)	0.001
Renal disease	1.17 (0.95-1.44)	0.150	1.07 (0.86-1.33)	0.544

NOAC: non-vitamin K oral anticoagulants, BMI: body mass index, MI: myocardial infarction, COPD: chronic obstructive pulmonary disease

Supplementary Table 8. Univariate and multivariable cox regression analysis for all-cause death in patients using warfarin versus NOACs

	Univariate analysis		Multivariable analysis	
	Crude HR (95% CI)	*P*-value	Adjusted HR (95% CI)	*P*-value
Type of anticoagulants				
Warfarin	1 (Ref)		1 (Ref)	
NOACs	0.94 (0.87-1.01)	0.075	0.87 (0.81-0.93)	< 0.001
Age, years				
< 65	1 (Ref)		1 (Ref)	
65-74	2.30 (2.01-2.64)	< 0.001	2.12 (1.85-2.43)	< 0.001
≥ 75	6.55 (5.81-7.38)	< 0.001	5.44 (4.81-6.16)	< 0.001
Male sex	0.70 (0.66-0.75)	< 0.001	1.00 (0.95-1.07)	0.894
BMI, kg/m^2	0.92 (0.91-0.93)	< 0.001	0.95 (0.94-0.96)	< 0.001
Hypertension	1.45 (1.35-1.56)	< 0.001	1.09 (1.01-1.18)	0.021
Diabetes mellitus	1.40 (1.32-1.49)	< 0.001	1.34 (1.26-1.42)	< 0.001
Dyslipidemia	0.78 (0.72-0.84)	< 0.001	0.71 (0.66-0.76)	< 0.001
Heart failure	1.57 (1.48-1.67)	< 0.001	1.30 (1.22-1.38)	< 0.001
Prior MI	1.57 (1.40-1.77)	< 0.001	1.33 (1.18-1.49)	< 0.001
Peripheral artery disease	1.19 (1.12-1.28)	< 0.001	1.01 (0.95-1.08)	0.677
COPD	1.63 (1.53-1.74)	< 0.001	1.31 (1.23-1.40)	< 0.001
Renal disease	2.11 (1.97-2.27)	< 0.001	1.60 (1.49-1.72)	< 0.001

NOAC: non-vitamin K oral anticoagulants, BMI: body mass index, MI: myocardial infarction, COPD: chronic obstructive pulmonary disease

Supplementary Table 9. Univariate and multivariable cox regression analysis for composite outcomes in patients using warfarin versus NOACs

	Univariate analysis		Multivariable analysis	
	Crude HR (95% CI)	*P*-value	Adjusted HR (95% CI)	*P*-value
Type of anticoagulants				
Warfarin	1 (Ref)		1 (Ref)	
NOACs	0.91 (0.86-0.97)	0.003	0.88 (0.83-0.93)	< 0.001
Age, years				
< 65	1 (Ref)		1 (Ref)	
65-74	1.46 (1.35-1.59)	< 0.001	1.36 (1.25-1.48)	< 0.001
≥ 75	2.75 (2.56-2.95)	< 0.001	2.35 (2.18-2.54)	< 0.001
Male sex	0.75 (0.72-0.79)	< 0.001	0.94 (0.90-0.99)	0.016
BMI, kg/m^2	0.96 (0.95-0.96)	< 0.001	0.97 (0.96-0.98)	< 0.001
Hypertension	1.33 (1.25-1.41)	< 0.001	1.08 (1.02-1.15)	0.007
Diabetes mellitus	1.34 (1.28-1.41)	< 0.001	1.28 (1.21-1.34)	< 0.001
Dyslipidemia	0.89 (0.84-0.95)	< 0.001	0.82 (0.77-0.88)	< 0.001
Heart failure	1.39 (1.33-1.46)	< 0.001	1.20 (1.15-1.27)	< 0.001
Prior MI	1.41 (1.28-1.56)	< 0.001	1.23 (1.12-1.36)	< 0.001
Peripheral artery disease	1.15 (1.09-1.21)	< 0.001	1.01 (0.96-1.07)	0.595
COPD	1.43 (1.35-1.51)	< 0.001	1.23 (1.16-1.30)	< 0.001
Renal disease	1.76 (1.66-1.87)	< 0.001	1.43 (1.34-1.52)	< 0.001

NOAC: non-vitamin K oral anticoagulants, BMI: body mass index, MI: myocardial infarction, COPD: chronic obstructive pulmonary disease

Supplementary Table 10. Number of outcomes of warfarin and low/standard-dose NOAC groups

	Warfarin (n = 2,889)		Low-dose NOAC (n = 9,238)		Standard-dose NOAC (n = 8,122)	
	Event (%)	IR	Event (%)	IR	Event (%)	IR
Ischemic stroke + systemic embolism	514 (17.8)	9.67	1,153 (14.4)	10.91	906 (13.2)	8.41
Major bleeding	281 (9.7)	4.99	637 (8.0)	5.77	389 (5.7)	3.42
All-cause death	915 (31.7)	15.28	2,540 (31.8)	21.69	1,214 (17.7)	10.31
Ischemic stroke + systemic embolism + GI bleeding + intracranial hemorrhage + all-cause death	1,362 (47.1)	27.01	3,622 (45.4)	36.14	2,106 (30.7)	20.17

IR: indicated incidence rate per 100 Person-year

Supplementary Table 11. Low- and standard-dose NOAC versus warfarin users: Hazard ratios of effectiveness and safety outcomes

	Ischemic stroke + systemic embolism		Major bleeding	
	aHR (95% CI)	*P*-value	aHR (95% CI)	*P*-value
Warfarin	1 (ref)		1 (ref)	
Low-dose	0.91 (0.82-1.01)	0.085	0.89 (0.77-1.03)	0.109
Standard-dose	0.81 (0.73-0.90)	< 0.001	0.66 (0.56-0.77)	< 0.001
	**All-cause death**		**Composite outcomes**	
	aHR (95% CI)	*P*-value	aHR (95% CI)	*P*-value
Warfarin	1 (ref)		1 (ref)	
Low-dose	0.96 (0.89-1.03)	0.280	0.97 (0.91-1.03)	0.308
Standard-dose	0.73 (0.67-0.79)	< 0.001	0.76 (0.71-0.82)	< 0.001
